# Complexes With Biologically Active Ligands. Part 9^1^ Metal Complexes of 5-Benzoylamino- and 5-(3-Nitrobenzoyl-Amino)-1,3,4-Thiadiazole-2-Sulfonamide as Carbonic Anhydrase Inhibitors

**DOI:** 10.1155/MBD.1997.1

**Published:** 1997

**Authors:** Andrei Jitianu, Marc A. llies, Fabrizio Briganti, Andrea Scozzafava, Claudiu T. Supuran

**Affiliations:** 1 “I.G. Murgulescu” Institute of Physical Chemistry Spl. Independentei, 202 Bucharest R-77208 Romania; 2 University of Bucharest Department of Chemistry Bd. Republicii, 13 Bucharest R-70346 Romania; 3 Università degli Studi Dipartimento di Chimica Laboratorio di Chimica Inorganica e Bioinorganica Via Gino Capponi 7 Florence I-50121 Italy

## Abstract

Complexes containing the anions of 5-benzoylamido-1,3,4-thiadiazole-2-sulfonamide and 5-(3-nitro-benzoylamido)-1,3,4-thiadiazole-2-sulfonamid as ligands, and V(IV); Cr(III); Fe(III); Co(II); Ni(II); Cu(II) and Ag(I) were synthesized and characterized by standard procedures (elemental analysis; IR, electronic, and EPR spectroscopy; TG, magnetic and conductimetric measurements). The original sulfonamides and their metal complexes are strong inhibitors of two carbonic anhydrase (CA) isozymes, CA I and II.


### COMPLEXES WITH BIOLOGICALLY ACTIVE LIGANDS. Part 9[1] METAL COMPLEXES OF 5-BENZOYLAMINO- AND 5-(3-NITROBENZOYL-AMINO)-1,3,4-THIADIAZOLE-2-SULFONAMIDE AS CARBONIC ANHYDRASE INHIBITORS

Andrei Jitianu[1,2], Marc A. Ilies[2], Fabrizio Briganti[3], Andrea Scozzafava[3] and Claudiu T. Supuran[3*]

[1] "I.G. Murgulescu" Institute of Physical Chemistry, Spl. Independentei, 202 R-77208 Bucharest, Roumania.
[2] University of Bucharest, Department of Chemistry, Bd. Republicii, 13, R-70346 Bucharest, Roumania.
[3] Università degli Studi, Dipartimento di Chimica, Laboratorio di Chimica Inorganica e Bioinorganica, Via Gino Capponi 7, I-50121, Florence, Italy.

**Abstract**: Complexes containing the anions of 5-benzoylamido-1,3,4-thiadiazole-2-sulfonamide and 5-(3-nitro-benzoylamido)-1,3,4-thiadiazole-2-sulfonamide as ligands, and V(IV); Cr(III); Fe(III); Co(II); Ni(II); Cu(II) and Ag(I) were synthesized and characterized by standard procedures (elemental analysis; IR, electronic, and EPR spectroscopy; TG, magnetic and conductimetric measurements). The original sulfonamides and their metal complexes are strong inhibitors of two carbonic anhydrase (CA) isozymes, CA I and II.

### Introduction

1,3,4-Thiadiazole-2-sulfonamide derivatives such as acetazolamide **1**, benzolamide **2** or chlorzolamide **3** are clinically used pharmacological agents, which owe their biological activity to the inhibition of the zinc enzyme carbonic anhydrase (CA, EC 4.2.1.1).[2-4] Since many isoforms of CA are presently known in higher vertebrates,[5] and since the physiological function for some of them is rather unclear, novel types of sulfonamide-[6,7] as well as non-sulfonamide[8] CA inhibitors are designed in order to select a series of compounds specific for the different CA isoenzymes, the final goal being the comprehension of the physiological functions of each of them and possibly their control. Among these, metal complexes of heterocyclic sulfonamides of type **1-3** exhibited valuable inhibitory properties against several CA isozymes.[9-11]

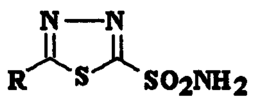

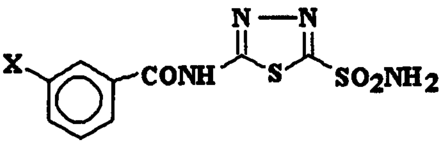

**1**: R = AcNH
**2**: R = $PhSO_2NH$
**3**: R =

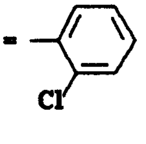

**4**: X = H
**5**: X = $NO_2$

Since there is a net discrimination of sulfonamide inhibitors as well as their metal complexes towards the rapid type CA isozymes (such as CA II and CA IV) versus the low type ones (such as CA I and III), our approach might lead to more specific inhibitors.[2,3,5]

In this paper we report the preparation of metal complexes of two new sulfonamide CA inhibitors, i.e., 5-benzoylamino-1,3,4-thiadiazole-2-sulfonamide **4**, and its 3-nitrophenyl- analogue **5**, which were recently reported by this group,[1] and which possess good CA inhibitory properties. The V(IV), Cr(III), Fe(III), Co(II), Ni(II), Cu(II) and Ag(I) complexes containing the conjugate bases of the two sulfonamides **4** and **5** were obtained and characterized by standard procedures (elemental analysis, IR, electronic, and EPR spectroscopy; TG, magnetic and conductimetric measurements). The new complexes possess good inhibitory properties against the red cell isozymes CA I and CA II, which are the predominant CAs is mammalian blood and secretory organs.[2,3,5]

### Materials and Methods

IR spectra of KBr pellets were recorded with a Specord M80 or Perkin-Elmer 16PC FTIR instruments, in the range 200-4000 $cm^{-1}$. Solution electronic spectra were recorded with a Specord M400 or Cary 3 spectrophotometers interfaced with a PC. Electronic spectra were obtained by the diffuse reflectance technique in MgO as a reference, with a Perkin Elmer Lambda 15 apparatus, in the range 200-900 $cm^{-1}$. Conductimetric measurements were done in 1 mM DMF solutions of the complex at 25°C with a Fisher conductimeter. EPR spectra of a crystalline powder were recorded on a Varian E-9 spectrometer at room temperature. The field was calibrated using crystalline diphenylpicrylhydrazyl (g = 2.0036). Magnetic susceptibility measurements were carried out at room temperature with a fully automated AZTEC DSM8 pendulum-type susceptometer. Mercury(II) tetrakis(thiocyanato)cobaltate(II) was used as a susceptibility standard. Corrections for the diamagnetism were estimated from Pascal's constants.[12] Elemental analyses were done by combustion for C,H,N with an automated Carlo Erba analyzer, and gravimetrically for the metal ions, and were ± 0.4% of the theoretical values. Thermogravimetric measurements were done in air, at a heating rate of 10°C/min., with a Perkin Elmer 3600 thermobalance.

Acetazolamide used in the enzymatic assay as standard was from Sigma. Sulfonamides **4** and **5** were prepared as described previously.[1] Metal salts and solvents were from Merck. Human CA I and CA II cDNAs were expressed in *Escherichia coli* strain BL21 (DE3) from the plasmids pACA/HCA I and pACA/HCA II (the two plasmids were a gift from Prof. Sven Lindskog, Umea University, Sweden). Cell growth conditions were those described by Lindskog's group,[13] and enzymes were purified by affinity chromatography according to the method of Khalifah et al.[14] Enzyme concentrations were determined spectrophotometrically at 280 nm, using a molar absorptivity of 49 $mM^{-1}$. $cm^{-1}$ for CA I and 54 $mM^{-1}.cm^{-1}$ for CA II, respectively, based on $M_r$ = 28.85 kDa for CA I, and 29.3 kDa for CA II, respectively.[15]

Initial rates of 4-nitrophenyl acetate hydrolysis were monitored spectrophotometrically, at 400 nm and 25°C, with a Cary 3 apparatus interfaced with an IBM compatible PC by the method of Pocker and Stone.[16]. $10^{-2}$ and $10^{-4}$ M solution of the substrate were prepared in anhydrous acetonitrile. A molar absorption coefficient $\varepsilon$ = 18,400 $M^{-1}.cm^{-1}$ was used for the 4-nitrophenolate formed by hydrolysis, in the conditions of the experiments (pH 7.80), as reported by Pocker and Stone.[16] Non-enzymatic hydrolysis rates were always subtracted from the observed rates. Duplicate experiments were done for each inhibitor, and the values reported throughout the paper are the averages of such results. $IC_{50}$ represents the molarity of inhibitor producing a 50% decrease of enzyme catalyzed hydrolysis of 4-nitrophenyl acetate.

### General procedure for the preparation of metal complexes 6-23

10 mMoles of sulfonamide **4** or **5** were suspended in 25 mL MeOH and the calculated amount of 1 N NaOH solution was added in order to obtain the monosodium salt $RSO_2NHNa$. This was treated thereafter with an aqueous solution of the metal salt ($AgNO_3$; chlorides for Cu(II), Co(II), and Ni(II), vanadyl and Cr(III) sulfate; Fe(III) perchlorate), working at molar ratios $M^{n+}$ : $RSO_2NH^-$ of 1:1 for the Ag(I) derivatives, 1:2 for the divalent metal ions and V(IV), 1:3 for the trivalent metal ions, and 1:4 for Co(II) and Cu(II). The reaction mixture was heated on a steam bath for 3 hours, then the precipitated complexes were filtered, thoroughly washed with cold water and alcohol. Crystallization was not done as the only solvents in which the complexes have good solubility are DMSO and DMF. The colored powders of complexes **6-23** melt with decomposition at temperatures higher than 325 °C.

### Results and Discussion

The metal complexes containing the conjugate bases of sulfonamides **4** and **5** and transition metal ions are shown in Table I, together with their elemental analysis data.

The newly prepared compounds, **6-23**, were also characterized by IR-, electronic and EPR spectroscopy, thermogravimetric (TG) and conductimetry. Some of these data are presented in Table II.

In the IR spectra of complexes **6-23**, the major modifications, as compared to the spectra of the sulfonamides **4** and **5** from which they were prepared, concern the two vibrations of the sulfonamido moieties, shifted by 5-10 $cm^{-1}$ (for the symmetric vibration) and 5-15 $cm^{-1}$ (for the asymmetric one), respectively, towards lower wavenumbers,[9-11] indicating that the deprotonated sulfonamido moieties of the ligands interacts with the metal ions, as well as the shift of the C=N vibration in the spectra of the complexes with 5-45 $cm^{-1}$ towards lower wavenumbers (except for compounds **13** and **22**) (Table II). The amide vibrations (the most intense such bands are at 1670-1680 $cm^{-1}$) of the complexes **6-19** appear unchanged as compared to those of the sulfonamides **4** and **5** (data not shown), suggesting that these moieties do not participate in coordination of the metal ions. Minor changes in the region 3100-3160 $cm^{-1}$ were also observed in the spectra of complexes **6-23**, as the bands present in the spectra of sulfonamides **4** and **5** are present in those of complexes, but they are not well resolved, and have a smaller intensity (data not shown). This is due to deprotonation of the $SO_2NH_2$ moiety and participation in the binding of cations.[1,9-11]

Table I: Prepared complexes **6-23**, containing the conjugate bases of sulfonamides **4** and **5**, and their elemental analysis data; bta stands for the sulfonamide deprotonated species of **4**, and nbta for the corresponding species of **5**.

| No. | Complex | Color | Yield (%) | %M[a] | Analysis (calculated/found) %C[b] | %H[b] | %N[b] |
|---|---|---|---|---|---|---|---|
| **6** | $[VO(bta)_2]$ | gray | 78 | 8.0/8.1 | 34.1/34.1 | 2.2/2.3 | 17.7/17.3 |
| **7** | $[Cr(bta)_3]$ | green | 82 | 5.7/5.4 | 35.9/35.5 | 2.3/2.1 | 18.6/18.4 |
| **8** | $[Fe(bta)_3]$ | brown | 90 | 6.1/6.0 | 35.8/35.5 | 2.3/2.3 | 18.5/18.3 |
| **9** | $[Co(bta)_2(OH_2)_2]$ | pink | 88 | 8.9/8.7 | 32.6/32.5 | 2.7/2.5 | 16.9/16.5 |
| **10** | $Na[Co(bta)_3]$ | pink | 71 | 6.2/6.1 | 34.8/35.1 | 2.2/2.0 | 18.0/17.6 |
| **11** | $[Ni(bta)_2(OH_2)_2]$ | green | 84 | 8.8/8.7 | 32.6/32.2 | 2.7/2.3 | 16.9/16.6 |
| **12** | $[Cu(bta)_2]$ | blue | 82 | 10.0/9.7 | 34.3/34.2 | 2.2/1.9 | 17.7/17.6 |
| **13** | $Na_2[Cu(bta)_4]$ | blue | 69 | 5.1/4.7 | 34.8/34.5 | 2.2/2.0 | 18.0/17.7 |
| **14** | $[Ag(bta)]$ | white | 95 | 27.6/27.1 | 27.6/27.7 | 1.8/1.9 | 14.3/13.9 |
| **15** | $[VO(nbta)_2]$ | gray | 82 | 7.0/7.1 | 29.8/29.5 | 1.6/1.5 | 19.3/19.0 |
| **16** | $[Cr(nbta)_3]$ | green | 67 | 5.0/4.7 | 31.2/31.6 | 1.7/1.4 | 20.2/19.9 |
| **17** | $[Fe(nbta)_3]$ | brown | 91 | 5.3/5.1 | 31.1/29.9 | 1.7/1.7 | 20.1/19.8 |
| **18** | $[Co(nbta)_2(OH_2)_2]$ | pink | 82 | 7.8/7.5 | 28.7/2843 | 2.1/2.2 | 18.6/18.5 |
| **19** | $Na[Co(nbta)_3]$ | pink | 85 | 5.5/5.4 | 30.3/30.1 | 2.2/2.0 | 19.6/19.3 |
| **20** | $[Ni(nbta)_2(OH_2)_2]$ | green | 94 | 7.8/7.4 | 28.7/28.4 | 2.1/2.3 | 18.6/18.3 |
| **21** | $[Cu(nbta)_2]$ | blue | 89 | 8.3/8.4 | 28.2/27.8 | 1.5/1.4 | 18.3/18.0 |
| **22** | $Na_2[Cu(nbta)_4]$ | blue | 75 | 4.4/4.2 | 30.4/30.0 | 1.6/1.6 | 19.7/19.4 |
| **23** | $[Ag(nbta)]$ | white | 95 | 24.7/24.5 | 24.7/24.8 | 1.3/1.0 | 16.0/15.8 |

[a]By gravimetry (calculated/found); [b]By combustion.

Solution electronic spectra of the complexes (Table II) prove the presence of the sulfonamido anions $RSO_2NH^-$ in the molecules of the new complexes **6-23**, as bathochromic and hyperchromic effects of the characteristic thiadiazole bands from 312-318 and 271-277 nm, respectively, are observed.[17] The two bands from the spectra of sulfonamides **4** and **5** undergo the same type of modifications when the spectra are registered in the presence of NaOH, when obviously the above mentioned anions are formed. This type of behavior was previously evidenced for other sulfonamide complexes which were prepared *via* the corresponding sodium salt.[9-11]

Thermogravimetric (TG) analysis (Table II) demonstrated the presence of two coordinated water molecules in the Co(II) complexes **9** and **18**, as well as the Ni(II) ones **11** and **20**. These water molecules are lost in a single step between 180-190 °C. The other prepared complexes did not show any weight loss under 240 °C. At higher temperatures they decomposed by a complete oxidation to NiO, as well as oxidation of the organic moieties present in their molecule (data not shown).

A detailed TG study was done for the Ni(II) complex **11**, which showed three steps of weight loss, described below:

$$[Ni(bta)_2(OH_2)_2] \xrightarrow{180°C} [Ni(bta)_2] + 2\,H_2O \qquad (1)$$

$$[Ni(bta)_2] \xrightarrow{245°C} 2\,C_6H_5CHO + \text{unidentified Ni(II) complex} \qquad (2)$$

$$\text{unidentified Ni(II) complex} \xrightarrow{250\text{-}700\ °C} NiO + CO_2 + SO_2 + N_2 + H_2O \qquad (3)$$

The first step is the one in which the two water molecules are lost (an exothermic process), as mentioned above. The anhydrous complex undergoes then a lost of benzaldehyde (step *2*) at 245 °C (endothermic process; weight loss, found: 34.1 %; calc. for two benzaldehyde molecules: 33.9 %), with formation of a highly unstable (unidentified) complex, which is further decomposed. Finally, the last step involves the complete oxidation of this last complex to NiO and gaseous compounds (step *3*).

Table II: Spectroscopic, thermogravimetric and conductimetric data for compounds **4-23**.

| **Comp.** | **IR Spectra**[a] ,cm$^{-1}$ $(SO_2)^s$ ; $(SO_2)^{as}$ | (C=N) | **Electronic Spectra**[b] λ (nm) (lgε) | **TG analysis**[c] calc./found | **Conductimetry**[d] $_M$ ($^{-1}$ x cm$^2$x mol$^{-1}$) |
|---|---|---|---|---|---|
| **4** | 1180; 1370 | 1610 | 318 (3.56); 271 (3.48) | e | 6 |
| **6** | 1170; 1360 | 1600 | 341 (3.62); 303 (4.03) | e | 9 |
| **7** | 1170; 1360 | 1600 | 339 (3.89); 301 (4.11) | e | 10 |
| **8** | 1160; 1350 | 1600 | 343 (3.80); 300 (4.08) | e | 7 |
| **9** | 1170; 1360 | 1600 | 341 (3.62); 276 (3.55) | 5.4/5.5[f] | 7 |
| **10** | 1180; 1360 | 1600 | 340 (3.69); 281 (3.52) | e | 183 |
| **11** | 1170; 1360 | 1565 | 340 (3.75); 286 (3.70) | 5.4/5.1[f] | 9 |
| **12** | 1180; 1350 | 1600 | 332 (3.61); 290 (3.66) | e | 11 |
| **13** | 1175; 1360 | 1610 | 340 (3.59); 308 (3.71) | e | 279 |
| **14** | 1170; 1360 | 1605 | 337 (3.58); 277 (3.52) | e | 11 |
| **5** | 1175; 1380 | 1620 | 312 (3.39); 256 (3.97) | e | 8 |
| **15** | 1170; 1360 | 1615 | 330 (3.48); 277 (4.01) | e | 14 |
| **16** | 1170; 1360 | 1610 | 340 (3.76); 284 (4.21) | e | 9 |
| **17** | 1160; 1360 | 1610 | 338 (3.72); 289 (4.09) | e | 12 |
| **18** | 1160; 1360 | 1615 | 345 (3.68); 284 (3.91) | 4.7/4.5[f] | 10 |
| **19** | 1170; 1355 | 1600 | 338 (3.98); 270 (4.10) | | 175 |
| **20** | 1170; 1360 | 1585 | 332 (3.58); 285 (3.87) | 4.8/4.6[f] | 13 |
| **21** | 1170; 1355 | 1610 | 346 (3.62); 290 (3.96) | e | 8 |
| **22** | 1170; 1350 | 1620 | 350 (3.43); 308 (4.10) | e | 250 |
| **23** | 1165; 1350 | 1600 | 338 (3.41); 269 (3.95) | e | 15 |

[a] In KBr; [b]In DMSO; [c]Weight loss between 130-200 °C; [d] $10^{-3}$ M solution, in DMF, at 25°C; [e] No weight loss seen under 250 °C; [f] Corresponding to two coordinated water molecules, lost at 180-190 °C.

Table III: Diffuse reflectance spectra, magnetic moments and proposed geometries for complexes **4-10**.

| **Complex** | **Electronic spectra** (, cm$^{-1}$)[a] | $_{eff}$ (BM)[b] | **Geometry** |
|---|---|---|---|
| **6** | 25,900; 15,500; 11,800(sh) | 1.85 | square pyramidal |
| **7** | 29,200; 23,700; 16,440 | 3.42 | octahedral |
| **8** | 24,650; 20,300; 10,600 | 5.77 | octahedral |
| **9** | 25,600; 20,500(sh); 15,600 | 4.89 | octahedral |
| **10** | 25,900; 20,800(sh); 15,900 | 4.90 | octahedral |
| **11** | 15,600; 11,400 | 3.48 | octahedral |
| **12** | 13,700 | 2.17 | square planar |
| **13** | 14,900 | 1.98 | distorted tetrahedral |
| **14** | c | d | linear |
| **15** | 25,800; 15,500; 11,700(sh) | 1.85 | square pyramidal |
| **16** | 29,400; 22,300; 17,200 | 3.40 | octahedral |
| **17** | 24,700; 20,800; 10,400 | 5.72 | octahedral |
| **18** | 25,900; 20,100(sh); 16,100 | 4.73 | octahedral |
| **19** | 26,500; 20,800(sh); 15,400 | 4.92 | octahedral |
| **20** | 16,100; 11,200 (sh) | 3.44 | octahedral |
| **21** | 14,200 | 2.20 | square planar |
| **22** | 13,800 | 1.73 | distorted tetrahedral |
| **23** | c | d | linear |

[a]In MgO as standard material; [b]At room temperature; [c] No transitions seen; [d] Diamagnetic.

Conductimetric data (Table II) showed the complexes **10** and **18** to behave as 1:1 electrolytes, the complexes **13** and **22** as 2:1 electrolytes, and all the other new complexes, as well as the sulfonamides from which they were prepared, as non-electrolytes.

The conjugate bases of the two sulfonamides **4** and **5**, used as ligands for the preparation of the complexes, probably act mono- or bidentately, as previously shown for the complexes containing main group metal ions.[1] When acting as monodentate ligands, the donor system is constituted by the sulfonamido nitrogen atom, whereas when bidentate, by the endocyclic N-3 and the sulfonamidic nitrogen.[1] It is quite probable that the two ligands have the same behavior in their complexes with transition metal ions, reported here. Monodentate behavior is present only for the two Cu(II) complexes **13** and **22**, whereas in all other compounds the ligands act bidentately. This behavior is supported by the spectroscopic, analytic and TG data presented above.

In Table III diffuse reflectance electronic spectra, magnetic susceptibility data at room temperature and the proposed geometries for the metal ions in the new complexes are presented.

Thus, the two vanadyl derivatives, **6** and **15**, show an electronic spectrum characteristic of V(IV) in square pyramidal geometry,[18] which is confirmed by the magnetic susceptibility data.[19] The Cr(III) and Fe(III) derivatives **7**, **8**, **16** and **17**, as well as the Ni(II) and Co(II) complexes are in octahedral geometry, as seen from both the electronic as well as magnetic data, typical for these ions in octahedral surrounding.[19-22] The two copper complexes in which the ligand acts monodentately, **13** and **22**, are probably in a distorted tetrahedral geometry, as they show only a broad band in the electronic spectrum, have magnetic moments under 2.00 BM, and a large signal in the EPR spectrum with $g_{\perp} = 1.93$ and $g_{\parallel} = 2.24$.[23-25]

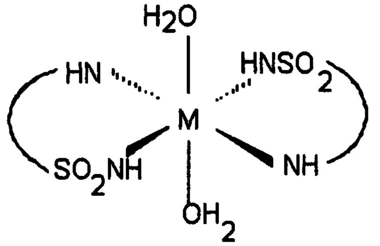

**9,11,18,20**

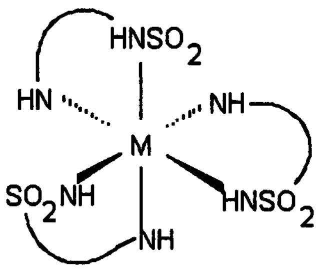

**7,8,10,16,17,19**

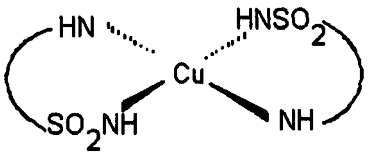

**12,21**

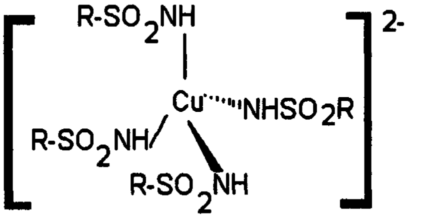

**13,22**

The other two copper derivatives, **12** and **21**, probably contain Cu(II) in square planar geometry,[24,25] as seen from the electronic spectroscopic and magnetic data of Table III. The Ag(I) derivatives **14** and **23** probably contain these ions in the linear geometry.[26]

Inhibition of isozymes CA I and II with the newly synthesized inhibitors as well as standard inhibitors acetazolamide **1** and benzolamide **2**, are shown in Table IV.

From the inhibition data of Table IV, it is obvious that the new complexes act as strong inhibitors of both isoymes, comparably with the standard (and very potent) CA inhibitor benzolamide, and much stronger ones than the other clinically used compound, acetazolamide.[2] No major differences were observed between the two sulfonamides **4** and **5**, except for the fact that the nitro-substituted compound **5** is slightly more active than **4** (a trend generally also manifested by the complexes containing the conjugate bases of these sulfonamides). This may be accounted on the acidification of the sulfonamido protons by the nitro moiety.[2,6c] The less efficient inhibitors were the V(IV), Cr(III) (it should be noted that compounds **7** and **16** are the first chromium complexes of sulfonamides ever reported) and Ni(II) derivatives, whereas cations such as Fe(III), Co(II), Cu(II) and Ag(I) led to the most active inhibitors.

Table IV: Biological activity data of sulfonamide CA inhibitors and their metal complexes ($IC_{50}$ -the mean of two different assays -represents the molarity of inhibitor producing a 50% decrease of enzyme specific activity for the *p*-nitrophenyl acetate hydrolysis reaction)[16].

| Compound | $IC_{50}$ (μM) CA I[a] | CA II[a] |
|---|---|---|
| **1 (acetazolamide)** | 90 | 1.10 |
| **2 (benzolamide)** | 12 | 0.10 |
| **4** | 9.0 | 0.15 |
| **5** | 8.0 | 0.09 |
| **6** | 10.5 | 0.10 |
| **7** | 12.4 | 0.15 |
| **8** | 3.5 | 0.09 |
| **9** | 1.5 | 0.06 |
| **10** | 2.9 | 0.05 |
| **11** | 8.0 | 0.13 |
| **12** | 4.3 | 0.05 |
| **13** | 3.2 | 0.04 |
| **14** | 1.8 | 0.03 |
| **15** | 10.0 | 0.08 |
| **16** | 12.5 | 0.09 |
| **17** | 2.5 | 0.03 |
| **18** | 1.8 | 0.04 |
| **19** | 1.4 | 0.04 |
| **20** | 7.6 | 0.11 |
| **21** | 1.5 | 0.02 |
| **22** | 1.8 | 0.01 |
| **23** | 2.4 | 0.03 |

[a]Human (cloned) isozyme;

Great differences between the two isozymes were also revealed, with CA II being much more susceptible to inhibition by sulfonamides and metal complexes, as compared to CA I. Although CA I is a relatively sulfonamide resistant enzyme,[2,3] it should be noted that several complexes reported here, such as **9**, **14**, **19**, and **21**, showed very good inhibition properties, although they also inhibited CA II quite potently, so that isozyme-specificity was not achieved yet with this class of inhibitors.

**Acknowledgments**: This research was financed in part by the EU grant ERB CIPDCT 940051. Thanks are addressed to Professor Sven Lindskog (Umea University, Sweden) for the gift of the plasmids expressing CA I and CAII.

**References**

1. Preceding part: A.Jitianu, M.A.Ilies, A.Scozzafava, C.T.Supuran, *Main Group Met. Chem.*, in press.
2. a) C.T.Supuran, "Carbonic anhydrase inhibitors", in "Carbonic Anhydrase and Modulation of Physiologic and Pathologic Processes in the Organism", I.Puscas Ed., Helicon, Timisoara **1994**, pp. 29-111; b) I.Puscas, C.T.Supuran, "Farmacologia clinica da ulcera peptica" in "Aparelho Digestivo", J.Coelho Ed., MEDSI, Rio de Janeiro, **1996**, pp. 1704-1734.
3. a) T.H.Maren, *Pharmacol.Rev.*, **1967**, *47*, 595-782; b) T.H.Maren, *J. Glaucoma*, **1995**, *4*, 49-62.
4. C.T.Supuran, C.W.Conroy, T.H.Maren, *Proteins*, **1997**, (in press).
5. D.Hewett-Emmett, R.E.Tashian, *Mol.Phylogenet.Evol.*, **1996**, *5*, 50-77.
6. a) C.T.Supuran, B.W.Clare, *Eur.J.Med.Chem.*, **1995**, *30* 687-696; b) C.T.Supuran, A.Nicolae, A.Popescu, *Eur.J. Med.Chem.*, **1996**, *31*, 431-438; c) C.T.Supuran, A.Popescu, M.Ilisiu, A.Costandache, M.D.Banciu, *Eur.J.Med.Chem.*, **1996**, *31*, 439-447; d) C.T.Supuran, C.W.Conroy, T.H.Maren, *Eur.J.Med.Chem.*, **1996**, *31*, 843-846; e) F.Briganti, R.Pierattelli, A.Scozzafava, C.T.Supuran, *Eur.J.Med.Chem.*, **1996**, *31*, (in press).

7. P.A. Boriack, D.W. Christianson, J. Kingery-Wood, G.M. Whitesides, *J.Med.Chem.*, **1995**, *38*, 2286-2291.
8. a) M.Rivière-Baudet, C.T.Supuran, *Main Group Met. Chem.*, **1996**, *19*, 579-584; b) A.Scozzafava, F.Briganti, C.T.Supuran, I.Fenesan, R.Popescu, V.Muresan, S.Farcas, *Main Group Met. Chem.*, **1996**, *19*, 503-507; c) C.T.Supuran, M.D.Banciu, G.Botez and A.T.Balaban, *Rev.Roum.Chim.*, **1992**, *37*, 1375-1383; d) C.T.Supuran, I.Saramet and M.D.Banciu, *Rev.Roum.Chim.*, **1995**, *40*, 1227-1232; e) C.T.Supuran, V.Muresan, R.Popescu and I.Fenesan, *Main Group Met. Chem.*, **1995**, *18*, 629-632; f) C.T.Supuran, S.V.Serves and P.V.Ioannou, *J.Inorg.Biochem.*, **1996**, *62*, 207-213.
9. a) G.Alzuet, S.Ferrer, J.Borras, C.T.Supuran, *Roum.Chem.Quart.Rev.*, **1994**, *2*, 283-300; b) C.T.Supuran, R.Stefan, G.Manole, I.Puscas, M.Andruh, *Rev.Roum.Chim.*, **1991**, *36*,1175-1179; c) C.T.Supuran, G.Manole, M.Andruh, *J.Inorg.Biochem.*, **1993**, *49*, 97-103; d) C.T.Supuran, *Rev.Roum. Chim.*, **1992**, *37*, 849-855
10. C.T.Supuran, A.Scozzfava, *J.Enzyme Inhibition*, **1997**, in press.
11. a) C.T.Supuran, *Rev.Roum.Chim.*, **1993**, *38*, 229-236; b) C.T.Supuran, *Roum.Chem.Quart.Rev.*, **1993**, *1*, 77-116; c) S.L.Sumalan, J.Casanova, G.Alzuet, J.Borras, A.Castiñeiras, C.T.Supuran, *J.Inorg.Biochem.*, **1996**, 62, 31-39; d) C.T.Supuran, G.L.Almajan, *Main Group Met. Chem.*, **1995**, *18*, 347-351; e) C.T.Supuran, *Metal Based Drugs*, **1995**, *2*, 327-330; f) C.T.Supuran, *Metal Based Drugs*, **1995**, *2*, 331-336; g) J.Borras, T.Cristea, C.T.Supuran, *Main Group Met. Chem.*, **1996**, *19*, 339-346; h) J.Borras, J Casanova, T.Cristea, A. Gheorghe, A. Scozzafava, C.T.Supuran, V. Tudor, *Metal Based Drugs*, **1996**, *3*, 143-148.
12. R.S.Drago, in "Physical Methods in Chemistry", W.B.Saunders & Co., London, **1977**, p. 411.
13.a) C.Forsman, G.Behravan, A.Osterman and B.H.Jonsson, *Acta Chem. Scand.*, **1988**, *B42*, 314-318; b) G.Behravan, P.Jonasson, B.H.Jonsson and S.Lindskog, *Eur.J.Biochem.*, **1991**, *198*, 589-592.
14. R.G.Khalifah, D.J.Strader, S.H.Bryant and S.M.Gibson, *Biochemistry*, **1977**, *16*, 2241-2247.
15. a) P.O.Nyman and S.Lindskog, *Biochim.Biophys.Acta*, **1964**, *85*, 141-151; b) L.E.Henderson, D.Henriksson and P.O.Nyman, *J.Biol.Chem.*, **1976**, *251*, 5457-5463.
16. Y.Pocker and J.T.Stone, *Biochemistry*, **1967**, *6*, 668-679.
17. a) C.T.Supuran, A.T.Balaban, M.D.Gheorghiu, A.Schiketanz, A.Dinculescu, and I. Puscas, *Rev.Roum. Chim.*, **1990**, *35*, 399-405; b) C.T.Supuran, A.Popescu and M.D.Banciu, *Rev.Roum. Chim.*, **1992**, *37*, 289-297.
18. C.J.Balhausen and H.Gray, *Inorg.Chem.*, **1962**, *1*, 111-115.
19. A.B.P.Lever, "Inorganic Electronic Specxtroscopy", 2-nd Edition, Elsevier, Amsterdam, **1984**, pp. 135-138.
20. B.N.Figgis and J.Lewis, *J.Chem.Soc.*, **1961**, 3188-3192.
21. L.Sacconi, F.Mani and A.Bencini, "Nickel", in "Comprehensive Coordination Chemistry", G.Wilkinson, R.Gillard and J.McCleverty Eds., Pergamon Press, Oxford, **1987**, Vol. 5, pp. 1-347.
22. L.Banci, A.Bencini, C.Benelli, D.Gatteschi and C.Zanchini, *Struct.Bonding*, **1982**, *52*, 37-79.
23. D.W.Smith, *Struct.Bonding*, **1978**, *35*, 88-117.
24. C.T.Supuran, C.I.Lepadatu, R.Olar, A.Meghea and M.Brezeanu, *Rev.Roum.Chim.*, **1993**, *38*, 1509-1517.
25. S. Ferrer, J.G.Haasnoot, R.A.G. de Graaf, J.Reedijk and J.Borras, *Inorg.Chim.Acta*, **1992**, *192*, 129-133.
26. C.T.Supuran, *Metal Based Drugs*, **1996**, *3*, 79-83.

**Received: November 22, 1996 - Accepted: January 7, 1997 - Received in revised camera-ready format: January 14, 1997**

